# Factors affecting psychological well-being: Evidence from two nationally representative surveys

**DOI:** 10.1371/journal.pone.0198638

**Published:** 2018-06-13

**Authors:** G. Oskrochi, Ahmed Bani-Mustafa, Y. Oskrochi

**Affiliations:** 1 College of Engineering, American University of the Middle East, Egaila, Kuwait; 2 Public Health Registrar, South London, United Kingdom; University of West London, UNITED KINGDOM

## Abstract

Financial status is thought to be an important determinant of psychological well-being. We investigate this relationship, and the effect of other factors, using a parametric mixed modelling approach for panel data, controlling the problem of unobservable heterogeneity.

Two nationally representative surveys, the British Household Panel Survey (BHPS) and the Understanding Society Survey (USS), were used to construct a unified data set which measured psychological well-being and associated factors using the 12-item General Health Questionnaire (GHQ-12). The GHQ-12 score for the head of the household was used as the dependant variable and its relationship with multiple independent demographic and financial status variables was investigated. Following assessment of growth curve characteristics with linear, curvilinear and higher-order polynomial modelling; several variance-covariance structures were tested to assess the error covariance structure of the longitudinal data. The random intercept and random slope were allowed to vary across participants, and methods such as natural splines and B-splines were used to improve the fit of some variables. Our final model demonstrated the most important variables affecting self-reported psychological well-being, as determined by GHQ-12, were perception and expectation of future financial situation and problems meeting household expenditure. Gender, age, marital status, number of children at home, highest qualification and job status were also significantly implicated. Unlike previous studies however we did not find that size of income was significant. These results provide further strong evidence of the impact that financial concerns have on self-reported measures of psychological well-being.

## Introduction and literature

There is a growing body of evidence implicating personal financial circumstances (PFC’s) and factors influencing PFC’s as determinants of individual health ([1], [2], [3]).

Some authors claim that this may be due to a number of indirect mechanisms and influences although their impact on health are equivocal ([4], [5]). Jones and Widman [5] suggest that these indirect mechanisms could constitute influences such as income inequality, relative deprivation, or other equally complex pathways.

This longitudinal study focuses on the impact of factors (direct or indirect) related to PFC on individual health and includes a large sample of head of the households (HoH) in Great Britain.

Econometricians have long investigated interdependent utility. Mean income levels in models of happiness have been used to control for inter-personal comparisons [6], whilst the relationship between well-being and income inequality [7] and life satisfaction and income distribution in Russia [8] have more recently been investigated.

These comparison effects are increasingly being recognised as important determinants of health. Jones and Wildman [5] noted that if lack of status causes consequent stress, then increasing working hours and general unhappiness, should be considered and accounted for.

Previous work has also identified that individuals who describe themselves as happy are more resilient to psychosomatic illnesses, less likely to take time off work, die prematurely, attempt suicide or seek psychological counselling ([9], [6]). Furthermore, if we accept the premise that health is vital for a flourishing life [10] then understanding the determinants of psychological well-being is equally vital.

## GHQ-12

The GL Assessment website defines GHQ as: “The General Health Questionnaire (GHQ) is a screening device for identifying minor psychiatric disorders in the general population and within community or non-psychiatric clinical settings such as primary care or general medical out-patients. Suitable for all ages from adolescent upwards–not children, it assesses the respondent’s current state and asks if that differs from his or her usual state. It is therefore sensitive to short-term psychiatric disorders but not to long-standing attributes of the respondent” (https://www.gl-assessment.co.uk/products/general-health-questionnaire-ghq).

The self-administered questionnaire focuses on two major areas; i) The inability to carry out normal functions; ii) The appearance of new and distressing phenomena.

It is available in different versions. GHQ-12 which has been used in this study is a quick, reliable and sensitive short form–ideal for research studies.

## Data

In this study, we explore factors affecting individual psychological well-being in Great Britain, considering the influence of financial situations and demographic characteristics. Our analysis is based on a sample drawn from two nationally surveyed datasets, the British Household Panel Survey (BHPS) and Understanding Society (US). Both BHPS and US contain information on financial situation, demographic information and individuals psychological well-being.

The BHPS is a nationally representative random sample longitudinal survey, carried out by the *Institute for Social and Economic Research*, of every adult in more than 5000 private households in Great Britain. Approximately 10,000 individuals were interviewed in waves (1–18) annually between 1991–2008. BHPS’ main objective was to further understand social and economic change at the individual and household level in Britain.

Sampling for subsequent waves consisted of all adults in all households which contained at least one member who was resident in a household interviewed at Wave 1. New eligibility for sample inclusion could occur between waves in the following ways:

- A baby born to an Original Sample Members (OSM);
- An OSM moves into a household with one or more new people;
- One or more new people move in with an OSM [11].

Interviews were sought with all resident household members aged 16 or over, therefore includes OSMs previously coded as children.

BHPS stopped at wave 18 in 2008, and its successor is Understanding Society (US).

Understanding Society (US) is another nationally representative random sample longitudinal survey of every adult in 40,000 UK households in Great Britain. US started in 2009 with interviewing all eligible members of the selected households either face-to-face or over the phone using Computer Assisted Interviewing (CAI).

The sample issued at wave 2 of US consisted of all members from the BHPS sample who were still active at Wave 18 and who had not refused consent to be issued as part of the Understanding Society sample [11]. As a result, wave one of US does not cover BHPS samples.

Our sample consists of a panel of 213,365 observations from 49,877 Head of the Households (HoH) who responded to any BHPS (1 to 18) or US (1 to 4) waves until end of 2012. Hence, the observational unit in our study is HoH’s as they have final responsibility for household financial situations and are therefore expected to be more affected psychologically.

## Key variables

### Dependent variable

Measure of psychological well-being derived from the General Health Questionnaire (GHQ) of the HoH is selected as the dependent variable.

The self-completion questionnaire component of the BHPS and US includes a reduced version of the GHQ [12].

The GHQ was originally developed to screen for psychiatric illness but has often been used as a proxy measure for psychological well-being. The shortened GHQ12 contains 12 individual elements which using a Likert [13] (1–4) scale, respondents state how they have recently felt regarding those elements [5].

The overall score ranges from 12 to 48 and lower value indicates good health. The Mean GHQ was calculated as the total GHQ divided by 12 to represent average GHQ of each individual. The mean GHQ therefore can be consider as a quantitative variable between 1 and 4. In this study the overall average of GHQ amongst 213365 data points is 1.938 (sd. 0.463).

The GHQ variable is a useful measure of health for determining the effect of income related variables because it measures psychological well-being and mental well-being which concentrates on broader components of psychiatric morbidity (particularly anxiety and depression). Detecting broad components of psychiatric morbidity is important if financial situation and deprivation are affecting individuals via stress or psychosocial mechanisms [14].

### Independent variables

Several variables are used in the empirical models to measure financial situation and financial expectation in the reference year (defined as the 12 months prior to the start of the interview time). The variables selected were listed below and the criteria for selection was based on the relevance and availability in both BHPS and US data. BHPS and US are supposed to be compatible by design. Generally, most of the information in BHPS somehow is available in US, but not necessarily under the same variable name or with the same format of coding. Therefore, many independent variables needed to be recoded to a unified version between BHPS and US.

### Scale variables

- The income variable gives monthly total household income. In the parametric mixed models, using the log of income to allows a non-linear relationship between health and income ([1], [15]) (INCOME_LN).
- Labour Income last month [FIMNL];
- Total Income last month [FIMN];
- Age in year at the date of the interview (AGE);
- Number of durable goods purchased (CD_All)
- Number of kids in the household (NKIDS)

Number of durable goods purchased controls extensively for recent purchases of durable goods. The variable indicates number of purchase of a colour television, video recorder, freezer, washing machine, tumble dryer, dishwasher, microwave, computer or CD-player, satellite, Cable TV, landline phone within the past years. The descriptive statistics of the quantitative variables are given in Table 1.

**Table 1:** Descriptive statistics of quantitative variables.

VARIABLE	NUMBER	MEAN	MEDIAN	S.D	MIN	MAX
**GHQ**	213,365	1.9383	1.83	0.463	1.0	4.0
**FIMNL**	213,315	1,028.8	510	1,500.2	-1500	72,055.4
**FIMN**	213,355	1422.2	1,166.7	1,313.8	-1500	72,176.5
**INCOME_LN**	213,363	6.93	7.14	1.78	-7.62	11.19
**CD_ALL**	212,791	8.12	9.0	2.26	0	12
**AGE**	213,365	50.64	49.0	17.38	16	100
**NKIDS**	213,361	0.544	0	0.95	0	10

In order to study the relationship between these independent variables and GHQ, pairwise correlation is calculated between the independent variables and GHQ and summarized in Table 2. Correlations between all independent variables were significantly and negatively correlated with GHQ except Number of Children (NKID) was significant and positively correlated with GHQ. The highest correlation was with FIMNL followed by FIMN. The least correlation was with age.

**Table 2:** Pairwise correlation matrix for all variables.

	GHQ	FIMNL	FIMN	INCOMELN	CD_ALL	AGE
**FIMNL**	-0.103					
**FIMN**	-0.08	0.799				
**INCOMELN**	-0.064	0.446	0.496			
**CD_ALL**	-0.054	0.29	0.276	0.188		
**AGE**	-0.019	-0.3	-0.118	-0.057	-0.195	
**NKIDS**	0.025	0.114	0.105	0.066	0.2	-0.416

### Categorical variables

- Gender (SEX);
- Marital status (MASTAT);
- Expected financial situation a year ahead (FISITX);
- Financial situation now (FISIT);
- Problem paying for housing over past year (XPHSDF);
- Highest Educational Qualification (QFEDHI)
- Job status (JBSTAT).

The descriptive statistics of the categorical variables are given in Table 3.

**Table 3:** Descriptive statistics for categorical variables including participant's characteristics.

Variable	Categories	n (%)
**Sex**	Male	118,596 (55.58%)
	Female	94,771 (44.42%)
**MASTAT**	Married or Couple	116,770 (54.73%)
	Single	41,039 (19.23%)
	Widowed, Divorced, Separated (**WDS**)	55,447 (25.99%)
**FISTITX**	Better than now (**Better**)	48,628 (22.79%)
	About the same (**AS**)	126,414 (59.25%)
	Worse than now (**Worse**)	31,729 (59.24%)
**FISIT**	Living comfortably or doing alright (**Good**)	132,152 (61.94%)
	Just about getting by (**Surviving**)	58,517 (27.43%)
	Finding it difficult (**Difficult**)	22,488 (10.54%)
**XPHSDF**	Problem paying for housing over past year	
	No	122,382 (57.36%)
	Yes	23,206 (10.88%)
	Missing	67,777 (31.76%)
**QFEDHI**	GCE A level and O Level, GSE A or O level, Scott 4–5, Apprenticeship (**GCSE**).	51,565 (24.17%)
**Highest Qualifications**	Higher degree and 1^st^ degree (**HDF**)	49,505 (23.20%)
	Teaching, Higher QF, Nursing, Other QF, Commercial QF (**THNOC**).	79,588 (37.30%)
	No Qualifications (**No_Q**)	31,120 (14.59%)
**JBSTATS**	Self-Employed, retired, FT student, Employed (**Stable**)	180,393 (84.55%)
	Temporary Employment (**TEMP**): Other, Maternity leave, Family care, Government training scheme, Unemployed, Temporary Employment	22,108 (10.36%)
	Long Term Sick (**LTS**)	10,836 (5.08%)

## Statistical methodology

Panel (longitudinal) data analysis is challenging as the responses are correlated in time. In addition, variance of such clustered data often changes with time. Disregarding the data clustered structure could lead to biased estimates of standard errors and potentially higher type I error ([16], [17]). Accordingly LMM has been suggested as an appropriate solution addressing the data clustered structure and modelling the complicated variance covariance structure of repeated measurements.

Adams et al [18] used panel data methods to control for unobserved heterogeneity in a similar circumstance. The issue of non-linearity of the relationship between wellbeing and income and misspecification in case of neglected non-linearity is discussed by Jones and Wildman [5]. Oskrochi et al [19], presented a model for analysing such panel data with multivariate linear mixed model with correlated random effects.

A number of studies have indicated the superiority of LMM to GLM or OLS models as the former produces error terms to control for the correlations resulting from the nature of the data clusters (individuals/and time), as described above.

Furthermore, Linear Mixed Model (LMM) allows tracking psychological well-being over time (Waves) for each HoH (treated as random effects in the model) by considering the impact of other time-invariant in the model [20]. It is well documented in the literature that LMM is superior to ordinary least squares (OLS/General Linear Models) because LMM theoretically models various variance covariance structures that control for potential dependency due to clustering effects, while OLS does not ([21], [22], [23], [24],[25]). An additional argument favouring the use of LMM is that it more accurately models error terms and controls Type I error rates [26].

Several models were tested to explore whether growth curves are linear or curvilinear with a two higher-order polynomial models, and if the rate of change in the dependent variable (GHQ) accelerated or decelerated across time. Several variance covariance structures were tested to assess the error covariance structure of the longitudinal data. The intercept and linear slope were permitted to vary across participants (household heads). On the other hand, as trajectories of GHQ decline/grow in time (*t*) can have different slopes and intercepts between households, LMM allows to treat the model coefficients as random (i.e. random slope, random intercept model).

Under the random coefficients modelling framework our model was progressively built with explanatory variables accommodating for different variance covariance structures.

As the true error structure is usually unknown, some comparing criterion are necessary to compare between models with different error structures, [20]. A backward stepwise procedure was adopted to select which variables and their interactions should remain in the final model, starting with a full model incorporating all fixed (with interaction between covariates) and random effects Decisions as to which variables to retain in the final model and the best variance covariance structure can be selected either by visual information, or more accurately based on comparisons of the differences in the Aikaike Information Criterion (AIC) given by (−2 *log likelihood* + 2*k*), as assessed by a chi-square test (p < 0.05), [27].

AIC measures the relative fit of competing models with different covariance patterns, where, *k* is the number of covariance parameters. This method sought to identify the “best” subset of influential factors on GHQ whilst simultaneously removing those variables that were redundant ([28], [29]). Furthermore, two methods of controlling for continuous confounding variables such as AGE were implemented in R; B-splines (bs; Basis for Polynomial Splines) and Natural splines (ns; Basis Matrix for Natural Cubic Splines).

The final model, fit using REML, was assessed using standard model diagnostic tools. All mixed-effects models were fitted using the “lme” function in “nlme” package in R 3.1.3 [30].

The general form of mixed effects model used is:

$$\begin{array}{l} {GHQ}_{ij}=\underset{fixed\;effect}{\underbrace{{(\beta }_{oo}+\beta_{10}t_{ij}+\beta_{2}t_{ij}^{2})}}+\underset{random\;effect}{\underbrace{\left(b_{0i}+b_{1i}t_{ij}\right)}}+(\beta_{3}AGE+\beta_{4}AGE^{2}+\beta_{5}AGE^{3})+\beta_{6}FISIT\\ \;+\beta_{7}FISITX+\beta_{8}FIMN+\beta_{9}FIMNL+\beta_{10}INCOME\_Ln+\beta_{11}XPHSDF\\ \;+\beta_{12}SEX+\beta_{13}MASTAT+\beta_{14}NKIDS+\beta_{15}JBSTAT+\beta_{16}QFEDHI\\ \;+\beta_{17}CD\_All+\beta_{18}SEX*MASTAT+\varepsilon_{ij} \end{array}$$

where *GHQ_ij_* is for head of the household (HoH) *i*, at time (year) *j*, *β_oo_* is the overall GHQ mean and *b*_0*i*_ is the HoH deviation from this mean, $\left[b_{0i}∼N(0,\sigma_{0}^{2})\right]$. Similarly, *β*_10_ is the mean growth/decline rate in GHQ and *b*_1*i*_ is the HoH well-being deviation from this growth/decline mean $\left(b_{1i}∼N(0,\sigma_{1}^{2}\right)$. *ϵ_ij_* are independent random errors for each household *i* and time *j*, $\epsilon_{ij}∼N(0,\sigma_{\varepsilon }^{2})$.

To test a nonlinear growth trajectory across time, other higher-order polynomial trends (i.e., quadratic (*β*_2_) was added to the model to improve model fit.

The linear slope suggests that the rate of growth/decline remains constant across time (i.e., a straight line), whereas the higher-order polynomial trends indicate that the growth rates might not be the same over time. A quadratic (second-order polynomial) change trajectory has no constant common slope (i.e., accelerate/decelerate over time) and consists of a single stationary point (i.e., peak/trough).

AGE, FISIT, FISITX, FIMN, FIMNL, INCOME, XPHSDF, SEX, MASTAT, NKIDS, JBSTAT, QFEDHI and CD_All are the covariates (defined above), which included in the model to evaluate their effect on the GHQ. *β*_3_,*β*_5_,…,*β*_17_ are the covariates coefficients respectively. Interaction between time and the potential predictors and confounding factors and interaction between SEX and Martial Status (MASTAT, *β*_18_) were also included in the model to test whether their effect varied across time points.

For identifying the most appropriate covariance structure, several possible autocorrelation structures were considered AR1, corARMA or moving average autocorrelation. Model selection based on Akaike Information Criterion (AIC) was used. corARMA covariance structures were selected and specified the best to our model based on AIC. Visual inspection of residual plots did not reveal any serious deviations from distributional assumptions, indicating that the fitted Linear Mixed Model was appropriate.

## Results

The best model (Table 4) was selected based on a stepwise selection retaining only the significant factors using the best variance covariance structure corARMA. In choosing the best mode, preference was given on smaller AIC values and fewer number of parameters to be estimated (parsimony). Striking a balance between these two aspects, a conditional polynomial model with heterogeneous corARMA(q = 3) covariance structure was found to be more appropriate for describing the growth/decline profile of GHQ.

**Table 4:** Model parameter estimates (SE).

**Independent Variables**	
**Fixed Effects**	Coefficients (Standard Error)
Intercept	2.215 (0.006)***
Time (Year)	2.722 (0.707)***
Time–Squared	-5.322 (0.47) ***
FISIT_Good	-0.296 (0.003)***
FISIT_Surviving	-0.194 (0.003)***
FISIT_difficult	Reference category
FISTIX_Better	-0.023 (0.002)***
FISTIX_Worse	0.057 (0.002)***
FISTIX_AS	Reference Category
FIMNL_log	NS
FIMN_log	NS
CD_All	NS
Income—log	NS
SEM_M	-0.071 (0.003)***
AGE	-4.401 (0.855)***
AGE-Squared	-2.702 (0.646)***
AGE-Cubed	11.749 (0.608)***
NKIDS	-0.012 (0.002)***
MASTAT_Single	0.017 (0.004)***
MASTAT_WDS	0.06 (0.004)***
MASTAT_Married or couple	Reference category
QFEDHI_GCSA	-0.03 (0.005)***
QFEDHI_HFD	-0.063 (0.006)***
QFEDHI_THONC	-0.048 (0.005)***
QFEDHI_No Qualification	Reference Category
JBSTAT_LTS	0.277 (0.005)***
JBSTAT_TEMP	0.099 (0.004)***
JBSTAT_Stable	Reference Category
**Random Components Standard deviation**			
Intercept	0.261***
Slope	0.0106***
Residuals	0.34
AIC	178481.4
R–Squared	0.5919

***p<0.001 NS: NOT significant factors are excluded from the model

The estimates of intercept, slope and quadratic slope of model were found to be significant and more realistic, with p-values less than 0.001.

The analysis suggests that, AGE, FISIT, FISITX, XPHSDF, SEX, MASTAT, NKIDS, QFEDHI and JBSTAT were significantly associated with GHQ. While FIMN, FIMNL, INCOME and CD_All were not found to be significantly associated with GHQ as illustrated in Table 4.

The estimate of the model intercept is 2.215, (SE = 0.006), and the slope (average change each year) is 2.722, (SE = 0.707). Both intercept and slop were found to be statistically significant in the model (p<0.001).

**Fig 1:**
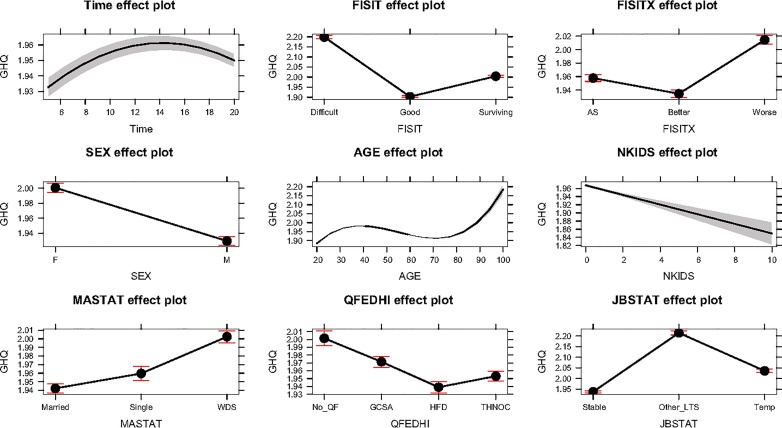
Mixed effect model significant covariates effects.

Time and time squared had a significant contribution in the model (p < 0.001), meaning that the effect of time on GHQ is nonlinear. The positive effect of linear growth (β = 2.72) and the negative effect of the quadratic term (β = -5.32) suggest that there is growth in GHQ score but this growth decelerates over time (concave down) (Time effect plot—Fig 1).

Random effects (intercept and slope) are also significant with standard deviations of 0.261 and 0.011 respectively, confirming that the initial status of the GHQ score and the growth/decline rates cannot be treated as fixed effects over time for each HoH.

Mean GHQ is a scale ranging from 1 to 4, with lower values indicating better perception of health. Our modelling suggests that as financial situation (FISIT) improves, GHQ scores lower (better health). The highest significant decrease in GHQ average score was for those who are “living comfortably or doing alright” (Good) (β = -0.296, p < 0.001), followed by those who “are surviving” (β = -0.194, p < 0.001), when compared to reference category of those who are “finding it difficult” (FISIT plot—Fig 1).

Financial expectations a year ahead (FISITX) show a similar trend. Those who expected a better financial year ahead had significantly less (better) GHQ average score (β = -0.023, p< 0.001) than those who expected no change (reference category). Those who expected a deterioration in their financial status had higher (poorer) GHQ (β = 0.057, p<0.001) scores compared to those who expected no change (FISITX effect plot—Fig 1.)

Income was not found to be significant and variability in income is explained mostly by job status and financial situations. Income was removed from the model for parsimony while job status and financial situations were included, using prior explained methodology.

With respect to demographic covariates; male HoH’s generally had significantly lower (better) GHQ score averages than females (β = -0.071, p<0.001). Age was also found to be nonlinear and significant with an increase (deterioration) in GHQ scores until the age of approximately 45, then declining until 75 before increasing again (Age effect plot—Fig 1). Number of children (NKIDS) reduced (improved) GHQ scores significantly for HoH’s (β = -0.012, p<0.001) per child.

Marital status (MASTAT plot–Fig 1) was also implicated; single HoH’s had a significantly higher (poorer) GHQ score (β = 0.017, p<0.001) when compared to married counterparts (reference category). Widowed, divorced, and separated (WDS) HoH’s also had significantly higher GHQ scores than married HoH’s (β = 0.056, p < 0.001).

In terms of educational attainment, HFD (β = -0.063), THNOC (β = -0.048) and GCSA (β = -0.030), denoting educated HoH’s, had significantly (p<0.001) lower GHQ scores than those with no qualifications (QFEDHI plot–Fig 1).

Finally, job status was also significantly (p<0.001) influential with a higher average (poorer) GHQ score for those deemed long term sick (LTS) (β = 0.277) compared to those with stable jobs (reference category). Temporary job holders (TEMP) also had significantly higher (poorer) GHQ scores (β = 0.099, p<0.001), than stable jobs (JBSTAT plot—Fig 1).

## Strengths and weaknesses

- Despite the complexity of fitting Linear mixed model, it is considered the most appropriate model for Panel data as it allows to model both the intercept and the slope to be fixed or random effects.
- Linear mixed model the most efficient model used to mode panel data with the most appropriate variance covariance (correlations between observations).
- The only potential weakness is that this analysis does not follow a particular cohort over time, to investigate the change in the effect to the same cohort.

## Discussion

We have demonstrated that both current as well as perceived future expectation of financial status have a significant impact on the psychological wellbeing of household heads (HoH’s). Other associated factors which were protective and promoted psychological well-being as determined by lower GHQ scores included male gender, married, presence of children, achieving higher educational qualifications and having a stable job.

Financial status and male gender have long been associated with mental-wellbeing scores and our findings are supported by other national level surveys ([31], [32]).

Our analysis however raises a nuance; in that current and future expectations of financial status are more important than absolute income in determining psychological well-being. The rationale for this could be explained by considering their definitions and connotations. Income is an absolute measuruement and is affected by “hard” adjustments such as local taxes and deductions, adjustments which the invdividual has no control over. Financial status however could be argued to be a more holsitic assessment with more individual control.

In assessing their financial status, an invidual may also consider additional factors such as their current or future level of debt or savings, expectations on current and future investments, spending habits and the earning potential of their partner or immediate family. In addition, financial status could also include “soft” influences such as perceived standard of living, future expenditure plans, and ability to afford what is desired (living comfortably). Furthermore, differences in life experiences may also impact financial status; those who have experienced previous hardships, either during childhood or adolesence may find that their current and future prospects are much better than what they have previously experienced, whereas those who have perhaps had much higher expectations previously may feel that they deserve more. Furthermore, an individuals percpetion of how much financial success matters as an indication of success can not be ignored, indeed, previous work has suggested that strong aspirations for financial success, to such an extent that it predominates over other life goals, has been associated with various pscyhological issues [33].

The implications are that consideration of income only when analysing financial effects on individuals is a crude measure at best, and erroenous at worst. Human perception of the world is defined by their perception of the future coloured with their experiences of the past, hence the use of a more holsitic variable which takes these into account would be a more appropriate method of analyisng financial effects.

Our findings also support previous work which demonstrate that marriage or the presence of a partner improve psychological wellbeing. Wilson and Oswald [34] in their longitudinal review of the impact of marriage on both physical and psychological health concluded that marriage reduced the risk of psychological illness and makes people happier and healthier. Interestingly they also concluded that although both partners enhjoy the benefits, there is evidence to show men may gain more than women, which could offer partial explanation of why men have better psychological well being scores.

Our results also show that the presence of children positively affects psychological wellbeing. The recent evidence in this area which demonstrates a relatively equivocal position with a slight predisposition towards a positive effect. The current concensus is that the effect of children is life-stage dependant and addtionally that it is not simply the number of children that are important, but rather the quality of the relationship between the child and their parents that influences parental psychological well-being [35]. Child age also appears to be an important factor, with parents of younger children reporting generally better psychological well-being scores [36]. Explanations range from the increasing autonomy of adolescent children [37], to less time spent with parents and questioning of parents rules and practices [38].

Job stability and security has also been previously identified to improve mental health and well-being, both in academic settings and in terms of policy advice [39]. A meta-analysis [40] demonstrated that mental health, as well as physical health, is significantly impacted by job insecurity. The authors argue that this finding is not surprising given that the anticipation of a fundamental and unwanted event (becoming unemployed in this case) is a source of psychological strain, and fits with the cnetral assumptions of stress theory.

Separatrely, De Witte [41] found that although job insecurity was a significant risk factor for psychological wellbeing in men, it was not so in women, offering poitential explanations using role theory and family situation, although noting that the limited number of women participants in the sutdy did not allow for meaningful conclusions.

Treating job security and insecurity as binary states and therefore applying the respective effects they have on psychological wellbeing as discrete events is also not appropriate. In their review of chronic job insecurity, Ferrie et al [42] showed that the psychological issues relating to periods of job insecurity persist even after job security has been regained. This suggests that the anxiety and stress associated with job insecurity is not easily forgotten, and that those who have experienced job insecurity, may always at some level be expecting to expeirence it again.

## Conclusion

This paper makes a several novel contributions to the literature. First and foremost, we have provided further evidence on the relationship between wellbeing and financial situation. Clear effects of the impact of financial situation on wellbeing for both men and women as the head of a household has been found. These results conform to the general literature, although this paper has explored a number of new points which we outline below.

We have demonstrated that the size of the income is not directly associated with wellbeing, while the financial situation and other controls have been accounted for in the model.

Perception of current and future financial situation and the prospect of a stable job is much more important than the size of the income on wellbeing.

We have highlighted a number of other factors which directly related to wellbeing; such as gender, age, education, number of children and marital status.

In term of the modelling several robust linear mixed models with linear or curvilinear growth curve methods and smoothing splines have been explored.

Interactions between factors affecting wellbeing are considered to assess whether important confounding factors are potentially being different for different groups.

The employed models accounted for individual heterogeneity and explored different patterns in order to produce far richer results by controlling for wellbeing dynamics.
